# Isolation of a persistent left superior vena cava using the circular array pulsed field ablation catheter

**DOI:** 10.1016/j.hroo.2025.05.013

**Published:** 2025-05-19

**Authors:** Yosuke Mizuno, Yohei Kikuchi, Keita Yoshiyama, Daiki Kumazawa, Kosuke Onodera, Takehiro Nomura, Kazuhiro Satomi, Kennosuke Yamashita

**Affiliations:** 1 Heart Rhythm Center, Department of Cardiovascular Medicine, Sendai Kosei Hospital, Tsutsumidori-Amamiyamachi, Aoba-ku, Sendai-shi, Miyagi, Japan; 2 Department of Cardiology, Tokyo Medical University, Nishi-Shinjuku, Shinjuku-ku, Tokyo, Japan

**Keywords:** Catheter ablation, Atrial fibrillation, Pulsed field ablation, Persistent left superior vena cava, Atrial tachycardia, Non-pulmonary vein trigger

**Key Findings**

▪ To our knowledge, this is the first comprehensive report on persistent left superior vena cava (PLSVC) isolation using the novel circular array-type pulsed field ablation (PFA) catheter, PulseSelect, highlighting its efficacy and safety.
▪ The over-the-wire, small-diameter design of PulseSelect makes it suitable for PLSVC isolation and enables precise application to the contact surface with the left atrium.
▪ PLSVC isolation by PulseSelect was successfully completed in all cases without complications. In contrast to conventional thermal ablation, PFA reduces the risk of damage to surrounding tissues.

## Introduction

A persistent left superior vena cava (PLSVC) is the most common thoracic venous anomaly in adults and provides a substrate for arrhythmogenesis.^1^ While thermal ablation to create a PLSVC isolation has been reported, it remains technically challenging and carries a significant risk of collateral damage to adjacent structures.^2^ A standardized approach has yet to be established.

Pulsed field ablation (PFA) is a novel non-thermal modality that induces irreversible electroporation of myocardial cells. This technique minimizes injury to adjacent structures during catheter ablation (CA), such as the esophagus and phrenic nerve (PN) by preventing apoptosis in surrounding tissues.

Although previous studies have shown the feasibility of PLSVC isolation using Penta-spline-type PFA catheters, no other catheter designs have been reported.^3^ Here, we evaluated the efficacy of the novel circular array PFA catheter, the PulseSelect (Medtronic, Minneapolis, MN), as a safe and effective alternative.

## Methods

Between September and December 2024, CA of atrial fibrillation (AF) was performed in 139 patients at our institution using the PulseSelect system. Among those, 3 patients (cases 1–3) had a PLSVC. All procedures were conducted under general anesthesia with propofol, rocuronium, and midazolam, assisted by the CARTO3 navigation system (Biosense Webster, Diamond Bar, CA) with a 14Fr bidirectional deflectable sheath (FlexCath Contour, Medtronic) and 10Fr long sheath for intra-cardiac echocardiography. While cases 1 and 2 were first sessions, only case 3 underwent a second session via the right internal jugular vein due to an interrupted inferior vena cava.

The pre- and post-ablation mapping, PLSVC sleeve assessment, and trigger site identification via induction testing were performed using a multielectrode mapping catheter (Octaray 3-3-3 mm, Biosense Webster). A novel circular array PFA catheter, the PulseSelect (9-electrode, 25 mm diameter, 3.75 mm electrode spacing, 3 mm electrode width, 9Fr), with an over-the-wire (OTW) design using a 0.032-inch J-wire, was used. Following the pulmonary vein isolation (PVI) with the PulseSelect, induction testing was conducted to identify the trigger site. As all 3 patients exhibited arrhythmogenic substrates requiring isolation, a PLSVC isolation was performed with the PulseSelect catheter. Post-isolation mapping of the PLSVC with the Octaray catheter confirmed the voltage elimination.

One or 7-day Holter monitoring was performed at 3 months post-procedure.

Case 3 was previously reported by Yamashita et al^4^ as an isolated case focusing on procedural feasibility via a superior approach.

This study was conducted in accordance with the Declaration of Helsinki. The study protocol was reviewed and approved by the Sendai Kosei Hospital Ethics Committee.

## Results

**Figure 1:**
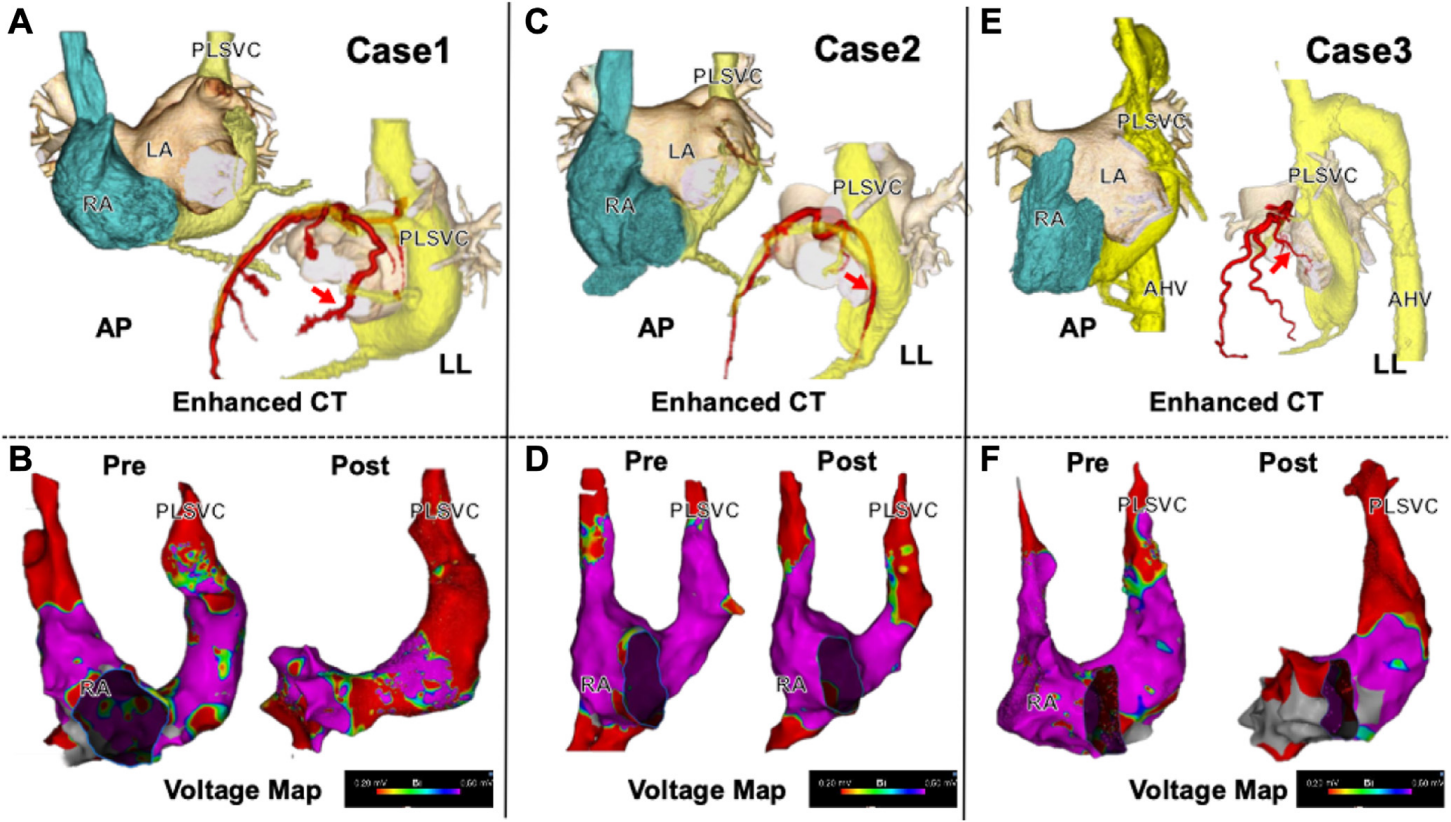
Three-dimensional reconstruction of the PLSVC by contrast-enhanced CT and pre-/post-isolation voltage maps of the PLSVC. **A, C, E:** Preprocedural contrast-enhanced CT images for each case. Red arrow: the main trunk of the left circumflex artery. **B, D, F:** Pre- and post-isolation voltage maps of the PLSVC obtained using the CARTO3 navigation system. AHV = Accessory hemiazygos vein; AP = anteroposterior; CT = computed tomography; LA = left atrium; LL = left lateral; PLSVC = persistent left superior vena cava; RA = right atrium.

The preprocedural contrast-enhanced computed tomography (CT) and bipolar voltage maps of the PLSVC pre- and post-isolation are shown in Figure 1. In the bipolar voltage pre-map during sinus rhythm, the low-voltage areas in the PLSVC were not notable under conventional threshold settings. However, after adjusting the bipolar voltage threshold, the PLSVC in case 2 exhibited a higher amplitude compared to the other cases (Supplemental Figure 1). The detailed clinical characteristics of each case are summarized in the Table.

**Table 1:** Clinical characteristics of each case

	Case 1	Case 2	Case 3
Age, y	74	42	63
Gender	Male	Female	Female
Height, cm	173.0	164.0	157.1
Body weight, kg	71.0	54.0	56.7
Body Mass Index, kg/m^2^	23.7	20.1	23.0
LV end-diastolic diameter, mm	49	40	47
LV end-systolic diameter, mm	31	29	26
LV ejection fraction, %	67	56	75
LA diameter, mm	39	28	35
Anomaly	PLSVC	PLSVC	Interrupted inferior vena cava PLSVC
Maximum diameter of the PLSVC by enhanced CT, mm	16	14	20
Distance from the PLSVC to the left circumflex artery by enhanced CT, mm	16.0	1.6	18.0
Coronary angiography	Yes	No	No
Type of AF	Persistent AF	Paroxysmal AF	Paroxysmal AF
Number of this CA sessions	First	First	Second
Details of previous session	None	None	PVI LA posterior wall isolation
Approach site for this CA	Right femoral vein	Right femoral vein	Right jugular vein
Details of this CA before the PLSVC isolation	PVI LA posterior wall isolation Lateral mitral isthmus block line	PVI	Re-right PVI
The type of arrhythmias associated with the PLSVC	Peri-mitral isthmus atrial tachycardia	Non-pulmonary vein trigger	Non-pulmonary vein trigger
Procedure times, min	209	67	115
Procedure time for the PLSVC isolation, min	15	8	16
Total number of applications during the procedure	74	63	55
Number of applications in the PLSVC	15	24	32
Isolation length from proximal to distal of the PLSVC, mm	57.4	54.6	61.5
Isolation area of the PLSVC, cm^2^	36.3	37	71.8
Fluoroscopy times, min	17.1	0.1	0.2
Fluoroscopy dose, mGy	36.60	0.04	0.21
Last follow-up duration post-CA, days	95	87	85
AF recurrence	No	Yes	No

AF = atrial fibrillation; CA = catheter ablation; CT = computed tomography; PLSVC = persistent left superior vena cava; PVI = pulmonary vein isolation; LA = left atrium; LV = left ventricular.

**Figure 2:**
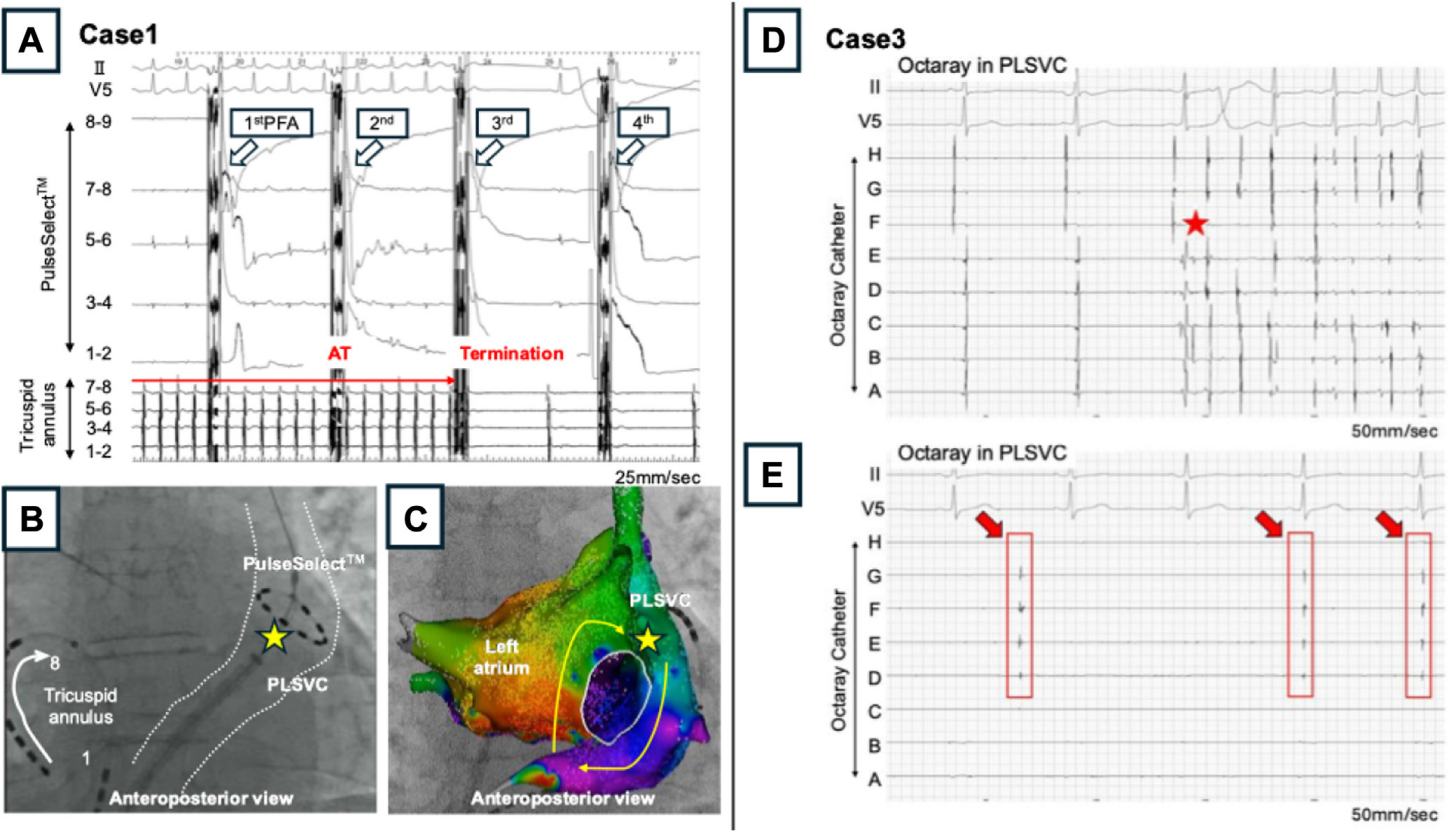
Details of atrial tachycardia in case 1 and PLSVC-originating non-PV trigger in case 3 Case 1 **A:** Intra-cardiac electrogram during AT termination. **B:** Fluoroscopy image during the PFA delivery to the PLSVC at the time the AT terminated. **C:** AT circuit (*yellow arrow*) visualized using a 3-dimensional navigation system. *Yellow star*: Position of electrode 5 on the PulseSelect catheter during the PFA delivery that terminated the AT. Case 3 D: Intra-cardiac electrogram recorded by the Octaray catheter during atrial fibrillation firing from the PLSVC (*red star*). E: Intra-cardiac electrogram showing dissociated potentials recorded in the isolated PLSVC using the Octaray catheter (*red arrow*). AT = atrial tachycardia; PFA = pulsed field ablation; PLSVC = persistent left vena cava.

In case 1, a mitral isthmus-dependent atrial tachycardia (AT) developed after the PVI. Non-pulmonary vein (PV) triggers from the PLSVC were identified in cases 2 and 3 (Figure 2D). The OTW catheter design enabled an easy delivery of the PulseSelect to the PLSVC via the J-wire, even in case 3, which required a superior approach.

The PLSVC applications followed the same protocol as the PVI, with 4 PFA deliveries as an application per targeted site. The PLSVC isolation was successful in all cases, with an average of 24 applications (range: 15–32), achieving a local electrogram elimination under sinus rhythm, and the exit block was confirmed by pacing from within the PLSVC using an Octaray catheter in each case. PLSVC isolation was performed to terminate AT and eliminate AF triggers, and ablation near the coronary sinus ostium was not necessary (Figure 1). Consequently, no conduction disturbances were observed during the procedure. Additionally, as the procedure was performed under general anesthesia, no coughing was induced by the PFA applications. And no clinically significant bradycardia occurred.

In case 1, activation mapping suggested a peri-mitral AT involving the PLSVC as part of the reentrant circuit (Figure 2C). Post-pacing interval from the PLSVC showed a 20 ms longer than the tachycardia cycle length, supporting its involvement. Despite posterior wall isolation and lateral mitral isthmus linear ablation with a radiofrequency catheter, the AT was not terminated and required additional PLSVC isolation. The peri-mitral AT was terminated after the 7th PFA application to the PLSVC (Figure 2A, B). In case 2, non-PV triggers originating from the PLSVC were identified during induction testing after standard PVI, and subsequent PLSVC isolation was performed. In case 3, after re-isolation of the right PV, induction testing similarly revealed non-PV triggers from the PLSVC, which were subsequently isolated (Figure 2D). After that, dissociated potentials from the PLSVC were observed post-isolation (Figure 2E).

No complications occurred during the procedures or follow-up period. Preprocedural CT confirmed the anatomical relationship between the PLSVC and coronary arteries (Figure 1). In case 2, nicorandil was administered because the main trunk of the left circumflex artery was nearby, and no coronary spasms occurred. PN capture was not observed intra-operatively due to muscle relaxants, and no diaphragmatic paralysis was seen on the post-operative chest X-rays.

AF recurred only in case 2. The mean follow-up was 89 days (range: 85–95). Pre-mapping during the re-do ablation revealed reconnection of the right inferior PV, which was subsequently re-isolated. In contrast, the PLSVC remained electrically isolated, confirming durable isolation (Supplemental Figure 2).

## Discussion

To the best of our knowledge, this is the first comprehensive evaluation of PLSVC isolation using the PulseSelect catheter. It expands on a prior single-case report by including a case series of 3 patients, along with detailed procedural data and follow-up outcomes, thereby offering broader insight into the feasibility, safety, and short-term efficacy of this approach.^4^

A PLSVC isolation requires electrical disconnection from both the LA and coronary sinus.^5^ However, conventional radiofrequency and cryoballoon ablation present specific challenges. Radiofrequency ablation requires multiple applications, increasing the risk of complications, and cryoballoon ablation is limited by the balloon size, making a complete occlusion difficult, while also presenting a risk of PN palsy. PFA can significantly mitigate these risks. However, a potential concern is coronary spasms, which depend on the anatomical proximity. Therefore, preprocedural CT imaging is crucial for accurately assessing the anatomical relationship.

Compared to the Farapulse (Boston Scientific, Boston, MA) used in previous studies,^3^ the PulseSelect is a relatively smaller diameter but more flexible circular array-type catheter, offering easier access to the distal PLSVC. Additionally, the PulseSelect enables flexion not only through sheath deflection but also via catheter deflection, allowing precise contact in the desired direction. This feature is particularly advantageous for PLSVC isolations, where ablation at the LA contact surface is necessary. In all 3 cases, PFA guided by the bipolar voltage successfully isolated the PLSVC starting from its distal portion. The design differences between PulseSelect and Farapulse may affect procedural maneuverability, but head-to-head comparison is necessary to determine clinical implications.

The mean number of applications for PLSVC isolation was 24, which may appear high. The post-ablation map showed an average PLSVC isolation area of 48.3 cm^2^ (range: 36.3–71.8) and length from proximal to distal of 57.8 mm (range: 54.6–61.5) (Table). This reflects the novelty of the procedure and the strategy of delivering multiple applications at each site to ensure complete isolation. It is possible that fewer applications could have achieved successful isolation. Further studies are warranted to establish the optimal lesion set and energy parameters for efficient and safe PLSVC isolation. Furthermore, database reports indicate that acute kidney injury and hemolysis have not been observed with the PulseSelect.^6^ While the application number and energy output may contribute, the PulseSelect may have an advantage in this regard. In our 3 cases, the total number of PFA applications increased for the PLSVC isolation; however, the mean pre-to-post procedural change in the serum creatinine levels was −0.07 mg/dl (range: −0.21 to 0.05), indicating no occurrence of kidney injury.

Given the small sample size and short follow-up duration, our findings are limited to early outcomes and should be interpreted with caution. This case series demonstrates the procedural feasibility and acute safety of PLSVC isolation using the PulseSelect catheter. Although our results are preliminary, they highlight the potential of this approach. Larger studies with extended follow-up are warranted to validate efficacy, lesion durability, and optimal ablation protocols.

## Conclusion

The novel circular array-type PFA catheter, the PulseSelect enabled a safe and effective PLSVC isolation. It allowed a precise intervention in the desired direction and reduced the complication risks as compared to thermal ablation. PulseSelect has potential as a preferred treatment for PLSVC isolations.
