# Supplementary material for: Isolation of a persistent left superior vena cava using the circular array pulsed field ablation catheter
Source: Heart Rhythm O2. 2025 May 19;6(8):1222–6. doi: 10.1016/j.hroo.2025.05.013 (PMC12411947; doi:10.1016/j.hroo.2025.05.013)
Supplement: Supplementary Figures 1 and 2 [file mmc1.docx]

**Supplementary Figure1**

Pre-isolation bipolar voltage maps of the PLSVC using the Octaray^TM^ catheter for each case. The bipolar voltage range was 0.1–2.5 mV, with Case 2 exhibiting higher voltage amplitudes compared to the other cases.

AP, anteroposterior; PA, posteroanterior; RA, right atrium; PLSVC, persistent left superior vena cava.

**Supplementary Figure2**

Bipolar voltage map of the PLSVC during sinus rhythm in Case 2, obtained three months after the initial session involving pulmonary vein isolation and PLSVC isolation. The map shows preserved electrical isolation of the PLSVC, with no indication of reconnection.
AP, anteroposterior; PA, posteroanterior; PLSVC, persistent left superior vena cava.
